# Phosphate Brain Energy Metabolism and Cognition in Alzheimer’s Disease: A Spectroscopy Study Using Whole-Brain Volume-Coil ^31^Phosphorus Magnetic Resonance Spectroscopy at 7Tesla

**DOI:** 10.3389/fnins.2021.641739

**Published:** 2021-04-06

**Authors:** Namrata Das, Jimin Ren, Jeffrey Spence, Sandra Bond Chapman

**Affiliations:** ^1^Center for BrainHealth, The University of Texas at Dallas, Dallas, TX, United States; ^2^Department of Radiology, Advanced Imaging Research Center, University of Texas Southwestern Medical Center, Dallas, TX, United States

**Keywords:** phosphate brain energy metabolism, Alzheimer’s disease, amnestic mild cognitive impairment, 31phosphorus magnetic resonance spectroscopy, adenosine triphosphate, mitochondria

## Abstract

**Introduction:**

Mitochondrial dysfunction is a neurometabolic hallmark signaling abnormal brain energy metabolism (BEM) targeted as a potential early marker of Alzheimer’s disease (AD). Advanced imaging technologies, such as ^31^phosphorus magnetic resonance spectroscopy (^31^P MRS) at ultra-high-field (UHF) magnetic strength 7T, provide sensitive phosphate-BEM (p-BEM) data with precision. The study’s first goal was to develop a methodology to measure phosphate energy and membrane metabolites simultaneously across the whole-brain using volume-coil ^31^P MRS at 7T in three groups-cognitively normal (CN), amnestic mild cognitive impairment (aMCI), and AD. The second aim investigated whether p-BEM markers in the four brain regions-frontal, temporal, parietal, and occipital were significantly different across the three groups. The final goal examined correspondence between the p-BEM markers and cognition in the three groups.

**Methods:**

Forty-one participants (CN = 15, aMCI = 15, AD = 11) were enrolled and completed cognitive assessment and scan. The cognitive domains included executive function (EF), memory, attention, visuospatial skills, and language. The p-BEM markers were measured using energy reserve index (PCr/t-ATP), energy consumption index (intracellular_Pi/t-ATP), metabolic state indicator (intracellular_Pi/PCr), and regulatory co-factors [magnesium (Mg^2+^) and intracellular pH].

**Results:**

Thirteen metabolites were measured simultaneously from the whole brain for all three group with high spectral resolution at UHF. In the aMCI group, a lower p-BEM was observed compared to CN group based on two markers, i.e., energy reserve (p = 0.009) and energy consumption (*p* = 0.05) indices; whereas in AD a significant increase was found in metabolic stress indicator (*p* = 0.007) and lower Mg^2+^ (*p* = 0.004) in the temporal lobes compared to aMCI using ANOVA between group analytical approach. Finally, using a linear mixed model, a significant positive correlation was found between Mg^2+^ and cognitive performance of memory (*p* = 0.013), EF (*p* = 0.023), and attention (*p* = 0.0003) in CN but not in aMCI or AD.

**Conclusion:**

To our knowledge, this is the first study to show that it is possible to measure p-BEM *in vivo* with precision at UHF across the three groups. Moreover, the findings suggest that p-BEM may be compromised in aMCI even before an AD diagnosis, which in future studies should explore to examine whether this energy crisis contributes to some of the earliest neuropathophysiologic changes in AD.

## Introduction

Neurometabolic abnormalities are now recognized as an important area for understanding the early neuropathophysiologic changes in Alzheimer’s disease (AD) of diverse etiology (Barzilai et al., 2012; Lourenço et al., 2015). Mitochondrial dysfunction at the root of many neurometabolic abnormalities is considered a high risk factor in AD (Yamaguchi et al., 1992; Albers and Flint, 2000; Crouch et al., 2005; Atamna and Frey, 2007). Research in the post-mortem brain of AD has shown that accumulation of beta-amyloid (Aβ) protein inside the mitochondria have the potential to alter structural and biochemical functions, accelerating the neurodegeneration process (Cottrell et al., 2001; Baloyannis, 2006; Lin and Beal, 2006). Mitochondria play an essential role in energy metabolism in all organs, especially the brain, due to their high energy requirements (Atamna and Frey, 2007). The observation of mitochondrial alteration in AD supports the postulation that disturbances in brain energy metabolism (BEM) may be a significant risk factor contributing to Aβ and tau deposition as individual’s progress from normal aging to transitory stages of AD, i.e., amnestic mild cognitive impairment (aMCI) and AD.

Magnetic resonance spectroscopy (MRS) offers a direct non-invasive imaging methodology to investigate mitochondrial function abnormalities by measuring the concentrations of phosphate energy and membrane metabolites, neuroinflammatory markers, and neurotransmitters in the brain (Ross and Sachdev, 2004). Specifically, ^31^phosphorus MRS (^31^P MRS) is used to measure phosphate energy [adenosine triphosphate (ATP), phosphocreatine (PCr), and inorganic phosphate (Pi-extracellular and intracellular)] and membrane phospholipid [phosphoethanolamine (PE), phosphocholine (PC), glycerophosphoethanolamine (GPE), and glycerophosphocholine (GPC)] metabolites along with the assessment of regulatory co-factors magnesium (Mg^2+^) and brain tissue pH which supports energy metabolism of the brain along with membrane synthesis and degradation. Recent work by Rijpma et al. (2018) using whole-brain volume-coil ^31^P MRS at 3T in thirty-one (31) participants with mild AD and healthy controls each showed increased PCr signal, PCr/Pi index, and pH in the retrosplenial cortex and hippocampus area of the temporal lobe in AD where early AD molecular changes are known to start. In contrast to alterations in phosphate BEM (p-BEM), the same study did not show any significant difference in the membrane phospholipid index measured using the PE, PC, GPE, and GPC in the brain regions of AD compared to healthy controls. Nonetheless, Rijpma’s work at 3-T in AD was one of the first study to apply a methodology to measure phosphate energy and membrane metabolites simultaneously from the whole-brain.

In addition to measuring phosphate metabolites through ^31^P signal intensities, the ^31^P-MRS also offers measurements of pH and Mg^2+^ concentration through ^31^P chemical shifts, based on the high sensitivity of Pi chemical shift δ(Pi) to Pi protonation/deprotonation and the dependence of ATP chemical shift difference δ(α-ATP)–δ(β-ATP) or δ_α–β_ on Mg^2+^ binding to ATP. Mg^2+^ plays a crucial role in regulation of mitochondrial functions as it stimulates over 300 enzymes (Adrasi et al., 2000). Prior research in *in vitro* studies using post-mortem human brains of AD reported reduced Mg^2+^ concentrations in vulnerable brain regions, especially in the hippocampus part of the temporal lobe (Adrasi et al., 2000; Cilliler et al., 2007; Slutsky et al., 2010). However, a knowledge gap exists in investigating the p-BEM, Mg^2+^, and pH abnormalities in aMCI, i.e., early transitory stage of the AD which is regarded as a heterogeneous and unstable condition as compared to AD and healthy aging. Therefore, it would be informative to include individuals in all three groups, i.e., healthy aging, aMCI, and AD to confirm and better understand those prior research findings from post-mortem AD brains, and to provide *in vivo* references for p-BEM alterations as an early marker of AD etiology and pathology responsible for neurobiological changes due to mitochondrial dysfunction. Whereas the cross-sectional research approach is motivated by evidence from *in vitro* studies of cell-line culture and post-mortem human brain (Atamna and Frey, 2007), there is an urgency in identifying early detectable and potentially treatable p-BEM abnormalities contributing to progressive neurodegeneration in aMCI, since this particular population is at greater risk for developing AD.

Prior to Rijpma’s work at 3T, a few studies investigated the relationship between p-BEM and cognition in AD with ^31^P data acquired from a single brain region at lower magnetic strength. Smith’s work using ^31^P MRS at 1.5-T in seventeen (17) mild to moderate AD and eight (8) healthy controls showed an inverse relationship of PCr/Pi index measured in the frontal lobe with a dementia rating scale (DRS), i.e., lower p-BEM marker was associated with higher DRS severity (Smith et al., 1995). Parallel to Smith’s work, another research by group Forlenza et al.’s (2005) using the same magnetic strength over the prefrontal cortex showed an inverse relationship between membrane phospholipid metabolite index with lower cognitive performance measured using the Cambridge Cognitive (CAMCOG) scale subtests in AD (*n* = 18) compared to healthy controls (*n* = 16). The CAMCOG scale included cognitive domains of memory, orientation, language, attention, praxis, calculation, abstract thinking, and visual perception. However, the main limitations at lower magnetic strength is the low spectral resolution of these metabolites, restricting the exploration of p-BEM-cognition correspondence in vulnerable brain regions like the temporal lobe compared to other parts of the brain in aMCI and AD. The low spectral resolution and detection sensitivity could be overcome by using ultra-high-field (UHF) magnetic strength at 7T for more accurate measurements of the phosphate metabolites.

At UHF magnetic strength 7T, the first aim of this research was to test the feasibility to acquire high quality whole-brain data for resolving phosphate brain energy and membrane metabolites from each voxel simultaneously across the three cohorts cognitively normal (CN) adults, aMCI, and mild AD using volume-coil ^31^P MRS. Our second aim was to investigate if p-BEM and membrane phospholipid markers differ across those three groups in the four regions-frontal, temporal, parietal, and occipital bilaterally. The p-BEM markers were measured using the three indices- energy reserve index (PCr/t-ATP), energy consumption index (intracellular_Pi/t-ATP), and metabolic state indicator (intracellular_Pi/PCr), along with regulatory co-factors – magnesium (Mg^2+^) and pH, separately (definitions of the three indices are cited in our previous publication; Das et al., 2020). Based on the prior study’s findings using either ^18^FDG-PET (Silverman et al., 2001; Mosconi et al., 2010; Mosconi, 2013; Ou et al., 2019) or ^31^P MRS (Rijpma et al., 2018), which supported reduced respective BEM (glucose or phosphate) in the temporal lobes in MCI and AD, we hypothesized that p-BEM markers-energy reserve, energy consumption indices, and regulatory co-factors (Mg^2+^ and pH), would be lower in the same vulnerable region of the brain and the same population, i.e., aMCI and AD when compared to CN group. The hypothesis of reduced p-BEM in the temporal lobe is the focus of this study because the brain depends on glucose consumption, which is regarded as the primary fuel to generate energy in the form of ATP (Mergenthaler et al., 2013). Moreover, prior 18FDG-PET studies have supported reduced glucose consumption in the temporal lobe, a region vulnerable to the early decline in AD pathology (Mosconi et al., 2010; Ou et al., 2019).

On the other hand, we anticipate that the metabolic state ratio, an indicator of metabolic stress, would be higher in aMCI and mild AD due to increased metabolic neurodegeneration compared to the CN group. Moreover, in congruence with study Rijpma et al.’s (2018), we proposed that the membrane phospholipid index would not be altered across the three groups.

The next aim explored the significant phosphate BEM markers-cognitive correspondence in all the groups. Prior studies using ^18^FDG-PET (Desgranges et al., 1998; Mosconi et al., 2005; Herholz, 2010; Ou et al., 2019) or ^31^P MRS at 1.5-T (Smith et al., 1995; Forlenza et al., 2005) have shown a BEM-cognition correspondence across the domain of memory, executive function (EF), attention, visuospatial perception, and language (Hanninen et al., 1996). Therefore, we postulated that there would be a significant association between lower sensitive p-BEM markers and co-factors (Mg^2+^ and pH) separately in the temporal lobe, a vulnerable brain region and lower cognitive performance across the three groups except for metabolic state indicator. The final goal was to compare the sensitivity and specificity of p-BEM markers and cognitive measures to reclassify the individuals in their respective groups correctly. Deep learning model/machine learning applications are rapidly developing to diagnose and predict who converts into AD. A recent application of the deep learning model was on 1,002 Alzheimer’s disease Neuroimaging Initiative (ADNI) participants to detect AD in 40 patients in an independent setting. The model showed a sensitivity of 100% and specificity of 82% in predicting the final diagnosis within an average timeline of 75.8 months before the AD diagnosis (Ding et al., 2019). Motivated by this evidence, we aimed to develop a prediction model for reclassifying aMCI and AD group by combining p-BEM markers with cognition.

## Materials and Methods

### Protocol Approvals and Consent

The research on human subjects was approved by the Institutional Review Board (IRB) of The University of Texas Southwestern Medical Center (UTSW#STU 062017-089) and The University of Texas at Dallas (UTD#18-73) to include individuals between the ages of 55-85years in three groups: CN adults, aMCI, and mild AD. Informed consent and HIPPA forms were signed as per the Committee on Human Experimentation’s under the Declaration of Helsinki revised in 1981 and useful clinical practice guidelines.

### Participants

A total of forty-one participants (15 CN, 15 aMCI, and 11 mild AD) were enrolled in the study using a phone screen, which included questions on demographics, medical history, medicine use, a neuroimaging screening questionnaire, and a memory-screen called Clinical Dementia Rating (CDR) scale. Subjects with a history of substance use, neurological disorders other than AD, psychiatric problems, metal in the body, or left-handers were excluded from the study. CN and aMCI were recruited from the DFW community based on ADNI criteria, and the classification was verified by a clinician and neuroscientist at Center for BrainHealth, UTD. Alzheimer’s patients with mild form were recruited from referral either through the local neurologist or from the DFW community after reviewing the individual’s medical records from the neurologist. All selected participants were right-handed, native English speakers with a minimum of 12 years of education. Irrespective of gender and ethnic factors, all eligible participants were invited to complete the cognitive screen and assessment.

#### Characterization of the Participants

The comprehensive ADNI criteria used for enrollment were 1) without subjective memory (CN) or with subjective memory (aMCI and AD) complaints; 2) Clinical Dementia Rating (CDR) scale: A score of zero (0) for CN, half (0.5) for aMCI, and a half or one (0.5 or 1) for mild AD (CDR, Morris, 1993); 3) mini mental status examination (MMSE) (Folstein et al., 1975): 24–30 for CN and aMCI and 20-26 for mild AD and; 4) objective memory status measured by logical memory subtest from Wechsler Memory Scale-III (WMS-III, Wechsler, 1997) with (a) delayed memory recall scores of ≥9 for 16 or more years of education, ≥5 for 8–15 years of educational and ≥3 for 0–7 years of education in CN, or (b) delayed memory recall scores of 8–11 for 16 or more years of education, 4–9 for 8–15 years of educational and 0-6 for 0-7 years of education for aMCI or (c) delayed memory recall scores of ≤8 for 16 or more years of education, ≤4 for 8–15 years of educational and ≤2 for 0—7 years of education for mild AD. In addition to ADNI criteria for the mild AD group, the diagnosis was confirmed by a neurologist. All participants were assessed for signs of depression using the long geriatric depression scale (GDS-long form) form (Yesavage et al., 1982). Individuals with no or mild depression with or without antidepressant medications were enrolled in the study.

### Cognitive Screening

A one-hour cognitive screen included vision and hearing test, vitals (blood pressure, pulse rate, weight, and height), and memory screens MMSE and logical memory subtest from Wechsler Memory Scale-III along with two questionnaires – 1. Lawton instrumental daily living activities, 2. Geriatric depression scale-Long form (GDS-long form) was completed at the Center for BrainHealth (CBH), a division of the University of Texas at Dallas (UTD). For eligibility on vision and hearing criteria, visual acuity of 20/50 and 40 dB at 1,000 HZ on the hearing test were the cut-off, respectively.

### Cognitive Assessment

Eligible participants on the phone and cognitive screens completed the cognitive assessment of 4-hours divided over 2 days to control for fatigue. The cognitive assessment included measures to investigate the domains of memory (episodic memory), executive function (complex abstraction, innovation, switching and inhibition, conceptual reasoning, working memory, and verbal fluency), attention, visuospatial skills, and language (Table 1). The demographics of the participants are presented in Table 2.

**TABLE 1:** Neurocognitive assessment battery administered across the three groups: cognitively normal, amnestic mild cognitive impairment (aMCI), and mild Alzheimer’s disease (AD).

Cognitive Domain	Measures	Description
**Executive Function**		
1. Complex abstraction	Test of Strategic Learning (TOSL) (Chapman et al., 2002) WAIS-III similarities (Wechsler, 1972)	Assess the ability to condense and synthesize complex information written as a summary from a short complex story. Scores represent a number of abstracted ideas. Assess the ability to think abstractly and to find similarities among words or ideas that may not appear to be similar on the surface.
2. Innovation	Test of Strategic Learning (TOSL) (Chapman et al., 2002)	Assess the ability to construct as many interpretations as possible from a complex short story to measure idea fluency.
3. Inhibition and switching	Trails B (Delis et al., 2001)	Assess the ability to alternate between a number and letter by drawing a continuous line.
4. Conceptual Reasoning	Delis-Kaplan executive function system (DKEFS) card sort (Delis et al., 2001)	Assess the ability to draw similarities between two sets of cards by drawing reasons behind the selection of cards.
5. Working Memory	Digit Span Backwards Test (WMS-III, Wechsler, 1997)	The ability to repeat a series of numbers backward.
6. Fluency: Verbal/Category	Controlled Oral Word Association (COWAT) (Benton et al., 1994; Spreen and Strauss, 1998)	Assess the ability to generate as many words starting with a particular alphabet or a category in one minute.
**Memory**		
Episodic memory	Memory for facts: Test of Strategic Learning (TOSL) (Chapman et al., 2002) California Verbal Learning Task (Petersen et al., 2001)	Assess the ability to recall details of a complex short story. Assess the ability to recall a list of sixteen (16) words in four categories immediately after the list was read followed by delayed recall after 20 min interval.
Attention	Selective Auditory learning task (Hanten et al., 2007) Digit Span Forward Task (WMS-III, Wechsler, 1997)	Assess the ability to focus and pay attention to high-priority stimulus, while simultaneously blocking or inhibiting unwanted or low-priority information. Assess the ability to pay attention and remember a series of numbers in the same sequence.
Language	Boston Naming Test (Kaplan et al., 1983)	Assess the ability of the individual to say the word associated with the object in the picture.
Visuospatial	Trails A (Delis et al., 2001)	The ability to visually search for numbers in ascending order and draw a continuous line assessing mental flexibility and processing speed.

**TABLE 2:** Demographics of the participant enrolled in the study.

	Cognitively normal (CN)	Amnestic Mild Cognitive Impairment (aMCI)	Mild Alzheimer’s disease (AD)
Gender	11 Females/4 Males	10 Females/5 Males	6 Females/5 Males
Age (mean ± SD)	63.47 ± 6.13	66.53 ± 6.74	71.73 ± 5.68*
Education	17.83 ± 2.91	17.33 ± 3.21	16.82 ± 3.68
Ethinicty (mean ± SD)	12 Caucasian/2 Asian/1 Hispanic	15 Caucasian	8 Caucasian/2 African American/1 Hispanic
Mini Mental Status Examination (mean ± SD)	29 ± 1.25	28.4 ± 1.404	25 ± 2.90
Clinical Dementia Rating (CDR) scale	0	0.5	0.5 or 1
Body Mass Index BMI (mean ± SD)	24.77 ± 3.19	26.43 ± 5.42	25.22 ± 4.89
Diabetes	1	0	1
Hypertension	1	6	5
Hyperlipidemia	4	7	5
Hypothyroidism	1	4	3

*p < 0.05.

### Whole-Brain p-BEM Metabolites Data Acquisition Using Volume-Coil ^31^P Magnetic Resonance Spectroscopy at 7T

**FIGURE 1:**
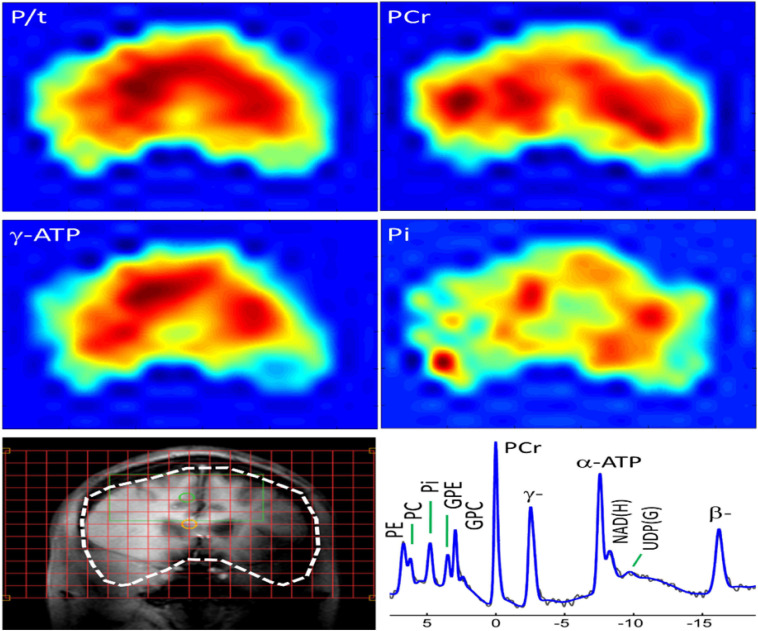
Whole-brain ^31^P MRS spectral displayed on coronal anatomical images. Total ^31^P signal from all metabolites; Color maps show the spatial distribution of specific metabolites.

**FIGURE 2:**
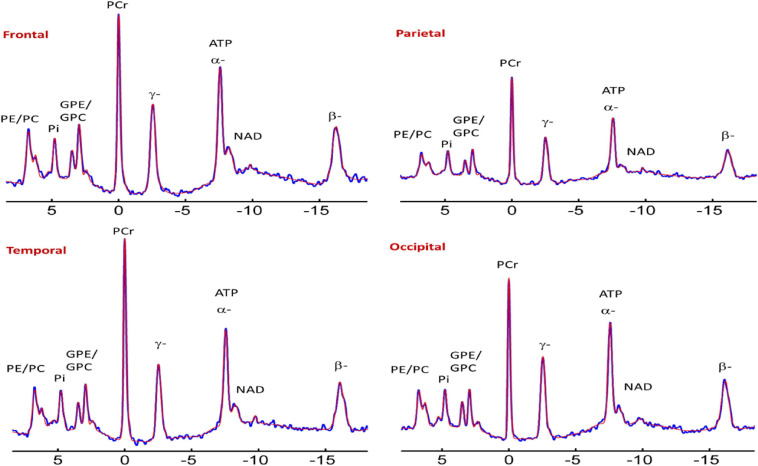
Representative spectra from the frontal, parietal, temporal, and occipital brain areas of a cognitively normal adult. Zero filling to 8,000 data points with 8 Hz vogit lineshape model applied for the display purpose. PE, phosphoethanolamine; PC, phosphocholine; Pi^*in*^, inorganic phosphate internal, respectively; GPE, glycerophosphoethanolamine; GPC, glycerophosphocholine; PCr, phosphocreatine; ATP forms: α, β, and δ adenosine triphosphate; NAD, nicotinamide adenine dinucleotide; UDPG, uridine diphosphate glucose.

The ^31^P-MRS data were acquired using a human MRI scanner system at 7T (Achieva, Philips Healthcare, Cleveland, OH, United States), in combination with a transmit/receive ^31^P birdcage volume coil of diameter 23 cm and length 10 cm (Gorten Center, Leiden University Medical Center, Netherlands). The ^31^P coil was inserted into a cylindrical NOVA ^1^H transmit/receive head coil for ^1^H shimming and imaging planning. The participants were positioned head-first, and supine with head posterior rest on a soft cushion and the head positioned in the center of the ^31^P RF coil. The data acquisitions included a non-localized ^31^P MRS survey scan with pulse-acquire sequence for evaluation of shimming quality on ^31^P spectrum, and a 3D MRS imaging scan at an in-plan resolution of 2 × 2 cm^2^ reconstructed to 1 × 1 cm^2^, slice thickness 2 cm, with 7–9 coronal slices depending on the participants’ head size along A-P direction. Other MRS parameters were TR = 0.5 s, TE 0.5 ms, number of average 12, sampling points 2 K, zero-filled to 4 K prior to FT scan time 39 min. Figures 1 and 2 shows the display of all the phosphorus metabolites.

### Phosphate BEM Metabolites Data Analysis

^31^P MRS raw data were preprocessed using the Philips software package SpecView on the scanner. All the brain slices were preprocessed for zero filings, apodization, Fourier transformation, and phase correction. For baseline correction and spectral fitting, the preprocessed data were post-processed using an in-house MATLAB program at UTSW. The fitting was based on the Voigt lineshape model (a combination of Gaussian and Lorentzian lineshape). Post-processed data was able to resolve thirteen (13) resonance phosphorus peaks of PCr, ATP (α-, β-, and γ-spins), nicotinamide adenine dinucleotide (total NAD), uridine diphosphate glucose (UDPG and its analogs), intracellular and extracellular Pi), five (5) phospholipid metabolites including PE, PC, GPE, GPC, and a macromolecular metabolite peak. The phosphate energy and membrane phospholipid metabolites were measured from each peak’s fitting integral. In contrast, the cellular pH and Mg^2+^ concentration were derived from the chemical shift measurements in region summed spectra for the four brain regions of interest- frontal, temporal, parietal, and occipital lobes. For each spectrum, the free intracellular magnesium (Mg^2+^) concentration was calculated using the chemical shift difference between α- and β-ATP (δ_α–β_ in ppm), whereas pH was calculated from the chemical shift of the corresponding Pi (internal) peaks (δ_Pi_, in ppm) in reference to PCr (δ_PCr_ = 0ppm) (Das et al., 2020). Total-ATP (t-ATP) signal was calculated by averaging the α-, β-, and γ-ATP resonances. For membrane phospholipid metabolite index/marker, phosphomonoesters (PMEs) was calculated by summation of PE and PC, whereas phosphodiesters (PDEs) by summation of GPE and GPC. Overall, the membrane phospholipid index was calculated by the ratio of PMEs/PDEs.

### Statistical Approach

#### Neurocognitive Measures, p-BEM Markers Along With Regulatory Co-Factors, and Membrane Phospholipid Markers Variations Across the Three Groups

We investigated the relationship between age, education, and gender with individual phosphate energy and membrane phospholipid metabolites. As we found significant inverse relationship of age and gender with the metabolites, all the p-BEM and membrane phospholipid metabolites were adjusted for age, education, and gender. Adjusted p-BEM data was used to calculate the ratios of PCr/t-ATP (reflective of energy reserve), Pi/t-ATP (reflective of energy consumption), Pi/PCr (reflective of a metabolic state), and regulatory co-factors (Mg^2+^ and pH) which were interpreted as BEM markers in the paper along with membrane phospholipid index (PMEs/PDEs) (for definition of the terminology refer to our previous publication Das et al., 2020). The BEM markers, membrane phospholipid indices, and regulatory co-factors were transformed into a shifted log scale to symmetrize their respective distributions and reduce high-leverage contributions in individual values regressions. Specifically, those with positive skew coefficients were transformed as log(*x* − *a*), and those with negative skew coefficients were transformed as −log(*a* − *x*), where “*x*” denotes the specific metabolite’s raw score for each participant and “*a*” denotes the metabolite-specific constant. In addition to the BEM metabolites, all the neurocognitive measures were also adjusted for age, education, and gender. Statistical analyses for the neurocognitive and spectroscopy data were analyzed using R studio 4.1.0 for windows. A one-way analysis of variance (ANOVA) between the group statistical package in R-studio was used to investigate the differences in the three groups with α = 0.01 for cognitive measures and α = 0.10 for BEM markers, given the exploratory nature of the study. *Post hoc* multiple between-group comparisons using Tukey at α = 0.05 for the familywise error rate was performed to identify significant mean differences between the groups.

#### Phosphate BEM and Regulatory Co-Factors Correspondence With Cognition Across the Three Groups

To navigate the next goal of the research, i.e., any p-BEM–cognition correspondences for which the transformed adjusted BEM and neurocognitive data were scaled to have common variance across the variables. General linear model was used to understand the effect of group and each of the markers or regulatory co-factors on cognition for each pairwise comparison. The model contains each of the indices or regulatory co-factor-by-group interaction on cognitive performance of EF, memory, attention, language, and visuospatial domains in the four brain regions-frontal, temporal, parietal, and occipital separately. All group tests and interaction tests were at α = 0.05.

The working linear model used in this work was written as:

$$\begin{array}{c} \text{y\_j }=\text{ b}0\text{ + b}1\;*\text{ I(group }=\text{ aMCI) }+\text{ b}2\;*\text{ I}(\text{group }+\text{ AD})+\\ b3\;*\text{ x\_j + b}4\;*\text{ x\_j }*\text{ I}(\text{group }+\text{ MCI})\;+\text{ b}5\;*\text{ x\_j}*\\ \text{I}(\text{group }=\text{ AD})\;+\text{ e\_j, for j}=\text{1, }...\text{, N subjects.} \end{array}$$

y_j represents the outcome or dependent variable, i.e., the cognitive performance for the *j*th subject. “*I*” is an indicator function (i.e., 1 if true; 0 otherwise), x_j is one of the indices or pH or Mg^2+^, e_j is the error term, and the covariates, x_j, are centered at their means. The inclusion of (indices/Mg^2+^/pH X group) and (indices/Mg^2+^/pH X mean differences between the group) interaction terms allowed for an in-depth investigation of all the probable effects on the cognitive outcome.

#### Predictive Model to Assess the Sensitivity, Specificity, Positive Predictive and Negative Predictive Value, and Accuracy of the Data Set

A principal component analysis was used for data reduction as an initial step to develop a predictive model using p-BEM makers and neurocognitive data (Supplementary Figure 1). The quadratic discriminant model was applied to the first two principal components that substantially explained much of the variance in the data. The discriminant model was trained using 10-fold cross-validation (CV) framework to obtain estimates of generalization error. For each of the 10-folds of the CV framework, 10% of the data was used as an internal hold-out test to predict the individuals in their respective groups, while the other 90% was used for training the model, including the principal component reduction.

## Results

### Neurocognitive Measures, p-BEM, Regulatory Co-Factors, and Membrane Phospholipid Markers Variations Across the Three Groups

#### Neurocognitive Measures Differences Across the Three Group

ANOVA between-group analysis across the three groups showed significant group effects in the five cognitive domains – EF, memory, attention, language, and visuospatial skills *z*-scores. In the executive function category all the subdomains: complex abstraction [similarities: *F*(2,38) = 38.87, *p* < 0.001]; innovation [TOSL: *F*(2,38) = 5.04, *p* = 0.01]; inhibition and switching [Trails B: *F*(2,38) = 50.28, *p* < 0.001]; conceptual reasoning [DKEFS sort: *F*(2,38) = 39.12, *p* < 0.001]; and fluency [verbal fluency: *F*(2,38) = 17.16, *p* < 0.001); category fluency-animals: *F*(2,38) = 10.38, *p* < 0.001] where significantly lower in mild AD compared to aMCI and CN. The subdomains of complex abstraction and conceptual reasoning performance were significantly reduced in aMCI along with mild AD but not in CN group. For the episodic memory performance [CVLT: immediate recall (*F*(2, 38) = 26.6, *p* < 0.001), short delay recall (*F*(2, 38) = 35.82, *p* < 0.001), long delay recall (*F*(2, 38) = 27.76, *p* < 0.001), intrusions (*F*(2, 38) = 8.60, *p* < 0.001)] was significantly lower in both mild AD and aMCI compared to CN. Similarly, attention domain measured by selective auditory learning test-Trail 1 (*F*(2,38) = 51.2, *p* < 0.001) and digit span forward test (*F*(2,38) = 4.077, *p* < 0.025); language tested using Boston Naming task (*F*(2,38) = 22.2, *p* < 0.001), and visuo-spatial skills assessed using Trails A (*F*(2,38) = 16.79, *p* < 0.001) was significantly lower in mild AD group only (Supplementary Table 1).

#### p-BEM Markers Variations Across the Three Groups

ANOVA between groups analysis showed three significant results. First, in aMCI and mild AD, the energy reserve index (PCr/t_ATP) [*F*(2,38) = 15.09, *p* < 0.001] in the temporal lobe was significantly lowered when compared to CN. Second, in aMCI energy consumption index (intracellular_Pi/t_ATP) [*F*(2,38) = 4.82, *p* = 0.01] of the temporal lobe was significantly lower compared to CN but not in mild AD. Finally, in temporal lobe of mild AD the metabolic state indicator (intracellular_Pi/PCr), [*F*(2,38) = 5.68, *p* = 0.007] was significantly higher when compared to aMCI and CN. The BEM markers in the other brain regions, i.e., frontal, parietal, and occipital were not significantly different across the groups.

The altered p-BEM markers in the temporal lobe in aMCI and mild AD prompted further exploration of which group means difference contributed to the significant effect using Tukey multiple comparisons adjustment. Overall, the results support that the aMCI group could be differentiated from the CN based on two BEM markers: energy reserve index (*t* = −0.88, *p*.adjusted Tukey = 0.009) and energy consumption index (*t* = −0.80, *p*.adjusted Tukey = 0.05), respectively. Next, the AD group could be differentiated from the aMCI cohort based on energy reserve index (*t* = −0.77, *p*.adjusted Tukey = 0.04) and metabolic state indicator (*t* = 1.15, *p*.adjusted Tukey = 0.007). Finally, all the p-BEM markers differentiated AD from CN [energy reserve index (*t* = −1.65, *p*.adjusted Tukey < 0.001), energy consumption index (*t* = −1.04, *p*.adjusted Tukey = 0.018), and metabolic state indicator (*t* = 0.95, *p*.adjusted Tukey = 0.03).

#### Regulatory Co-Factors Variations Across the Three Groups

Regulatory co-factor intracellular Mg^2+^ was significantly lower in the temporal lobe in mild AD compared to aMCI and CN [*F*(2,38) = 6.48, *p* = 0.0038]. Moreover, Tukey multiple comparisons adjustment in this model showed a similar pattern as the metabolic state indictor reported above, pointing to a significant mean difference in two specific group comparisons, i.e., AD to CN (*t* = −1.03, *p*.adusted tukey = 0.015) and AD to aMCI (*t* = −1.19, *p*.adjusted tukey = 0.004), however, differences between aMCI and CN failed to reach significance (*t* = 0.16, *p*.adjusted tukey = 0.87). The results from other brain regions were insignificant.

##### Membrane Phospholipid Marker (PMEs/PDEs) Variations Across the Three Groups

**FIGURE 3:**
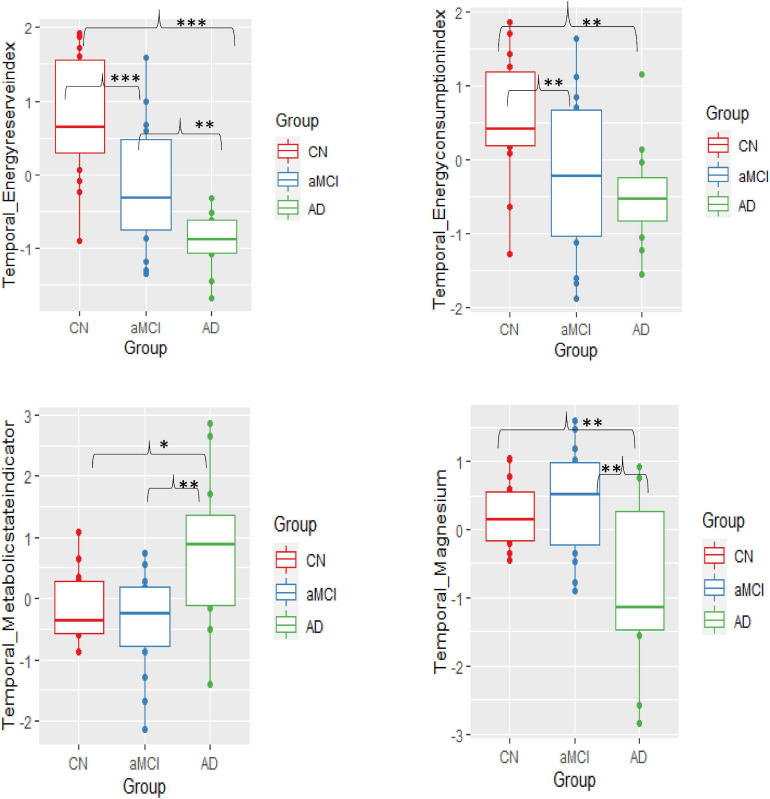
Group mean differences of BEM markers – energy reserve index, energy consumption index, metabolic state, and intracellular pH in the temporal lobe across the three groups-cognitively normal (CN), amnestic mild cognitive impairment (aMCI), and mild Alzheimer’s disease (AD) (significant differences *p* = 0.10*, *p* = 0.01**, *p*≤0.001***).

The membrane phospholipid marker (PMEs/PDEs) failed to reach significance across groups in the four brain regions separately [frontal: *F*(2,38) = 1.76, *p* = 0.18; temporal: *F*(2,38) = 0.91, *p* = 0.41; parietal: *F*(2,38) = 1.85, *p* = 0.17; occipital: *F*(2,38) = 2.12, *p* = 0.13]. Table 3 and Figure 3 summarizes the findings.

**TABLE 3:** ANOVA analysis of p-BEM markers, Regulatory co-factors (Magnesium and pH), and membrane phospholipid markers in the four brain regions-frontal, temporal, parietal, and occipital across the three cohorts: cognitively normal (CN) adults, amnestic mild cognitive impairment (aMCI), and mild Alzheimer’s disease (AD) (*indicates those *F* tests which satisfy FDR = 0.10). Tukey *post hoc* analysis for the significant group difference.

Independent variables	Brain region	ANOVA results *df* = (2,38) F-statistics p-value df = (2,38)		*Post hoc* Tukey multiple comparisons		
				MCI to CN mean difference (p-value)	AD to CN mean difference (p-value)	AD to MCI mean difference (p-value)
**BEM indices**						
Energy reserve index: PCr/t-ATP	Frontal Temporal Parietal Occipital	0.042 15.09 1.57 0.76	0.96 <0.001*** 0.22 0.47	0.11(0.96) −0.88(0.009) −0.63(0.20) −0.01(0.999)	0.07(0.99) −1.65(<0.001) −0.23(0.83) −0.44(0.52)	-0.04(0.99) -0.77(0.04) 0.40(0.56) -0.43(0.53)
Energy consumption index: intracellular Pi/t-ATP	Frontal Temporal Parietal Occipital	0.106 4.82 1.87 0.003	0.9 0.01* 0.17 0.997	−0.08(0.98) −0.80(0.05) −0.44(0.45) 0.03(0.99)	−0.19(0.89) −1.04(0.018) 0.29(0.73) 0.03(0.99)	-0.11(0.96) -0.24(0.79) 0.73(0.16) 0.09(0.99)
Extracellular Pi/t-ATP	Frontal Temporal Parietal Occipital	3.01 2.06 0.202 2.12	0.06* 0.14 0.82 0.13	0.43(0.44) −0.71(0.12) 0.22(0.82) −0.35(0.59)	0.93(0.05) −0.32(0.69) 0.04(0.99) 0.44(0.49)	0.50(0.40) 0.40(0.56) -0.18(0.89) 0.80(0.11)
Metabolic state indicator: intracellular_Pi/PCr	Frontal Temporal Parietal Occipital	0.126 5.68 0.76 0.516	0.88 0.007** 0.47 0.60	−0.07(0.98) −0.21(0.80) 0.001(0.999) −0.07(0.98)	−0.20(0.87) 0.95(0.03) 0.44(0.52) 0.32(0.71)	-0.13(0.95) 1.15(0.007) 0.44(0.53) 0.39(0.60)
Extracellular_Pi/PCr	Frontal Temporal Parietal Occipital	3.27 3.44 0.47 2.53	0.049* 0.042* 0.63 0.09*	0.47(0.38) −0.08(0.97) 0.34(0.63) −0.32(0.64)	0.96(0.04) 0.83(0.08) 0.07(0.98) 0.54(0.34)	0.49(0.40) 0.91(0.05) -0.27(0.78) 0.86(0.076)
**Regulatory co-factor**						
Intracellular pH	Frontal Temporal Parietal Occipital	1.34 0.25 0.27 3.40	0.26 0.78 0.77 0.04*	−0.17(0.24) −0.03(0.99) 0.27(0.75) −0.36(0.56)	−0.17(0.91) 0.23(0.83) 0.09(0.97) −0.98(0.03)	0.42(0.53) 0.27(0.79) -0.17(0.91) -0.62(0.24)
Intracellular magnesium(Mg^2+^)	Frontal Temporal Parietal Occipital	0.61 6.48 2.03 1.50	0.55 0.0038** 0.14 0.24	−0.34(0.63) 0.16(0.87) −0.14(0.92) −0.03(0.99)	0.05(0.99) −1.03(0.015) 0.61(0.27) −0.61(0.27)	0.39(0.60) -1.19(0.004) 0.75(0.14) -0.59(0.30)
**Membrane phospholipid indices**						
PMEs/PDEs	Frontal Temporal Parietal Occipital	1.76 0.91 1.85 2.12	0.18 0.41 0.17 0.13	−0.11(0.95) −0.27(0.75) 0.61(0.22) −0.73(0.11)	0.59(0.29) 0.27(0.78) 0.61(0.27) −0.32(0.69)	0.69(0.18) 0.54(0.38) 0.01(1.00) 0.41(0.55)

*p < 0.10, **p < 0.01, ***p.0.001.

### Phosphate BEM-Cognition Correspondence Across the Three Groups

**FIGURE 4:**
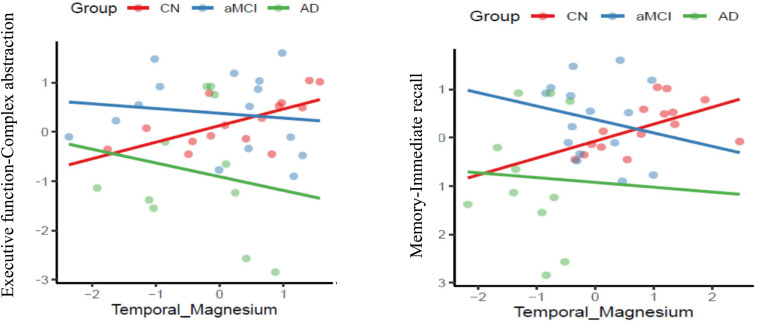
Association of co-factor magnesium (Mg^2+^) in the temporal brain region with cognitive performance domains of executive function and memory respectively across the three groups-cognitively normal (CN-red color), amnestic mild cognitive impairment (aMCI-blue color) and mild Alzheimer’s diseases (AD-green color) (*p* ≤ 0.05).

The final step was to investigate the association of significant or sensitive temporal BEM markers – energy reserve index, energy consumption index, metabolic state indicator, and intracellular Mg^2+^ with cognitive domains of executive function, memory, attention, language, and visuospatial skills across the three groups. Overall, a linear mixed model was used to explore the BEM-cognition correspondence. Figure 4, Table 4, and Supplementary Table 2.

**TABLE 4:** Main effect of BEM and regulatory co-factor (Magnesium^2+^) interaction with the clinical group on cognitive performance of domains of executive function, memory, attention, visuospatial skills, and language.

Region of interest temporal lobe indices or regulatory co-factor and clinical group effect on cognition	ANOVA between-group results		

	F(df = 2,38)	p-value	Mean squares
**Energy reserve index (PCr/t-ATP)**			
Executive function Verbal fluency A inhibition and switching-Trails B	4.6979 7.4030	0.0156* 0.0020**	2.443 1.629
Visuospatial domain Trails A	3.9816	0.0277*	1.688
**Energy consumption index (intracellular_Pi/t_ATP)**			
Executive function Verbal fluency (A)	3.2148	0.0522*	1.8657
Memory Episodic memory California Verbal Learning Task (CVLT) 1. List A-immediate recall 2. Repetitions	3.2879 3.8632	0.0491* 0.0305*	1.2066 2.7135
**Metabolic state indicator (intracellular_Pi/PCr)**			
Executive function Innovation-TOSL	4.6303	0.0164*	3.3063
Memory Episodic memory California Verbal Learning Task (CVLT) Recognition	5.7276	0.0070**	4.1099
**Regulatory co-factor-Intracellular Magnesium (Mg^2+^)**			
Executive function Complex abstraction-TOSL	3.2135	0.0523*	2.9203
**Memory**			
Episodic memory	3.6597	0.036*	1.4347
California Verbal Learning Task (CVLT) List A-immediate recall			
**Attention**			
Strategic Auditory attention test Trail 1 Trail 2	8.087 3.723	0.0012** 0.034*	1.7004 2.3072

Significance *p* < 0.05*, *p* < 0.01**, *p* < 0.001***.

#### Temporal Lobe Energy Reserve Index-Cognition Correspondence

In aMCI, the group-by-temporal lobe energy reserve index interaction effect revealed a significant positive association with EF – verbal fluency *z*-scores [*F* (2,38) = 4.6979, *p* = 0.0156] in aMCI group (*t* = 1.70, *p* = 0.10), whereas a negative correlation trend was found in mild AD (*t* = −1.98, *p* = 0.06) compared to CN. Moreover, the mild AD group contributed to the significant positive correlation effect by the interaction of energy reserve index and group on EF-inhibition and switching subdomain measured using Trails B *z*-scores [*F* (2, 38) = 7.4030, *p* = 0.002] compared to aMCI (*t* = −1.18, *p* = 0.25), a negative association with AD (*t* = 3.20, *p* = 0.003) compared to CN.

#### Temporal Lobe Energy Consumption Index-Cognition Correspondence

In aMCI, the group-by- temporal lobe energy consumption index was strongly associated significantly with EF-verbal fluency *z*-scores [*F*(2,38) = 3.2148, MS = 1.8657, *p* = 0.05] in AD group compared to aMCI in negative direction (*t* = −2.36, *p* = 0.023). In addition CN group, the main effect of the interaction was also strongly associated with episodic memory-immediate list memory recall *z*-scores [*F*(2,38) = 3.2879, MS = 1.2066, *p* = 0.0491] whereas the interaction coefficients between the groups where not significant.

#### Temporal Lobe Metabolic State Indicator-Cognition Correspondence

The group-by-temporal lobe metabolic state indicator effect was significantly inversely associated with EF-innovation subdomain *z*-scores [*F*(2,38) = 4.6303, MS = 3.3063, *p* = 0.0164]. The main effect was attributed by the CN as positive association between the independent and dependent variables compared to aMCI (*t* = 2.68, *p* = 0.01) and mild AD (*t* = 2.94, *p* = 0.006). Additionally to the above results, in aMCI a significant inverse relationship was shown with episodic memory- recognition subdomain [*F* (2, 38) = 5.7276, MS = 4.1099, *p* < 0.007] compared to CN (*t* = −2.65, *p* = 0.011), where a positive correlation was found in AD compared to MCI (*t* = 3.08, *p* = 0.004).

#### Temporal Lobe Intracellular Mg^2+^-Cognition Correspondence

The main effect of interaction of group-temporal lobe intracellular Mg^2+^ on EF-complex abstraction [*F*(2,38) = 3.2135, MS = 2.92, *p* = 0.05] compared to aMCI (*t* = −2.38, *p* = 0.023)] and mild AD (*t* = −2.39, *p* = 0.02) compared to CN group. Similarly episodic memory domain-immediate recall [*F*(2,38) = 3.6597, MS = 1,4347, *p* = 0.036] interaction effect was contributed by the aMCI (*t* = −2.62, *p* = 0.013) and mild AD (*t* = −2.45, *p* = 0.02) compared to CN group. Finally, the interaction of group-temporal lobe intracellular Mg^2+^ on attentiotrail 1 domain [*F*(2,38) = 8.087, MS = 1.7004, *p* = 0.0012] was associated with aMCI (*t* = −3.99, *p* = 0.003) and mild AD (*t* = −3.39, *p* = 0.002) compared to CN group.

### Predictive Model Results

The principal component reduction and the quadratic discriminant model utilized both the MRS values and neurocognitive data set. For each of the 10 folds, the model was trained on 90% of the data set and tested on hold-out 10% of the data. The cross-validation error was 0.1704 with seven (7) misclassifications out of the 41 individuals data set used in the analysis with a standard error of 0.0513. The respective sensitivity was 73.3% for CN (11 of 15), 80% for aMCI (12 of 15), and 100% for AD (11 of 11), whereas specificity was 88.5% for CN, 84.6% for aMCI, and 100% for AD. The accuracy was 82.9% with the standard error was 0.051. Table 5 summarizes all the predictive model results.

**TABLE 5:** Prediction model using the discriminant model for classifying the individuals into the group accurately a) confusion matrix b) sensitivity, specificity, positive predictive value (PPV), and negative predictive value (NPV).

a) Confusion matrix				
	**Group**			
**Prediction**		**CN**	**aMCI**	**AD**

	CN	11	3	0
	aMCI	4	12	0
	AD	0	0	11

b) Sensitivity, specificity, positive predictive value (PPV), and negative predictive value (NPV).				

**Group**	**Sensitivity**	**Specificity**	**PPV**	**NPV**

CN	0.733	0.885	0.786	0.852
aMCI	0.800	0.846	0.750	0.880
AD	1	1	1	1

## Discussion

In this pilot work using whole-brain volume-coil ^31^P MRS at UHF magnetic strength 7T, the first aim was to test the feasibility of measuring phosphate energy and membrane metabolites simultaneously across the three cohorts: CN adults, aMCI, and mild Alzheimer’s disease (AD) with precision. With an improved SNR ratio, we supported our hypothesis that at UHF 7T, a distinct peaks of thirteen (13) phosphorus metabolites can be simultaneously acquired from the whole brain consistently across the three populations. Moreover, this research adds on aMCI a transitory, unstable, and heterogeneous group where some are at risk for AD to the existing p-BEM’s work that investigated the difference between AD and healthy controls at 3T (Rijpma et al., 2018). aMCI group inclusion in this research fills the gap in knowledge of phosphate BEM alterations in early stage before an AD diagnosis is clinically apparent. In essence, the inclusion of all three groups suggested the viability of studying whole-brain p-BEM application, a window to investigate mitochondrial dysfunction theory which is postulated as an early neurometabolic marker in the pathology of AD from *in vitro* studies (Atamna and Frey, 2007) to *in vivo* using ^31^P MRS.

The next goal was to investigate whether p-BEM markers and regulatory co-factors (Mg^2+^ and pH) could detect group-level differences across three groups in the four brain regions: frontal, temporal, parietal, and occipital. The work’s main aim was to examine if measurable p-BEM markers could distinguish the between-group difference of aMCI from CN, one of the most significant challenges facing to develop effective AD diagnosis and treatment. The crucial finding was that the aMCI group was differentiated from CN based on two phosphate BEM markers, specifically, those represented by the energy reserve index and the energy consumption index. Moreover, these significant differences in the two phosphate BEM markers were localized to the temporal lobes bilaterally, the region most commonly vulnerable to early changes in AD’s transitory stages (Mosconi et al., 2010; Ou et al., 2019). Furthermore, this research adds to the growing body of evidence on the neurobiological factors at the molecular level in terms of energy metabolism are altered in aMCI and can be detected which are the prime target group for clinical interventions. In sum, the lower energy reserve and energy consumption indices in the aMCI as compared to the CN group suggests that lower phosphate BEM may represent a cellular energy crisis in aMCI, potentially leading to the neurodegeneration process as the disease progress.

Our findings of reduced p-BEM metabolism in the at-risk population could be explained by two theories-mitochondrial dysfunction (Atamna and Frey, 2007) or glucose BEM hypometabolism (Mosconi et al., 2010; Yin et al., 2016), respectively. Mitochondria aids in the replenishing PCr and Pi stores to maintain a continuous supply of energy in the form of ATP. The creatine kinase enzyme controls the release of energy or vice-versa from PCr and Pi (Hettling and van Beek, 2011). Prior work in the post-mortem AD brain has shown decreased creatine kinase activity levels compared to individuals free from any neurodegenerative disorder (David et al., 1998; Aksenov et al., 2000). Thereby, we infer the reduced creatine kinase may potentially disrupt the stores of energy reserve and energy consumption metabolites – PCr and Pi, respectively. Parallel to mitochondrial dysfunction theory, the glucose BEM hypometabolism hypothesis directly affects energy reserve and consumption in the brain as glucose is the brain’s primary energy production source (Atamna and Frey, 2007; Mosconi et al., 2010). However, an unanswered question is whether the upstream mitochondrial abnormalities of energy metabolism alter glucose metabolism or if the reverse is true and disruptions in glucose metabolism lead to phosphate BEM changes. Although we cannot answer the direction of the relationship of these markers and glucose metabolism, the study complement prior work on glucose metabolism and *in vitro* mitochondrial dysfunction in aMCI and AD to explore the order of changes further in the *in vivo* setting. This finding is the first evidence suggesting that energy reserve and consumption indices may provide a viable way to measure and identify early p-BEM etiology to explain pathophysiological brain change during a transitory stage of those at-risk for developing AD, namely individuals with aMCI. Therefore, we speculated that the compromised phosphate BEM mechanism leads to an energy crisis, which might be an early biomarker of a degrading brain moving toward dementia.

Additionally, we were also interested in investigating which p-BEM markers and regulatory co-factors could separate mild AD from aMCI. Notably, the p-BEM markers-energy reserve index, metabolic state indicator, and regulatory co-factor Mg^2+^, mainly in the temporal brain region, separated the two groups. The results supported the hypothesis that the energy reserve index and Mg^2+^ would be lower in mild AD than aMCI. In contrast, the metabolic state indicator marker was higher in mild AD than aMCI. A metabolic state indicator ratio was previously used as a marker to assess mitochondrial function (Chance et al., 1981; Pettegrew et al., 1997; Valkovič et al., 2019). Compared to healthy controls, a higher metabolic status indicated that an organ (e.g., brain or heart) is in metabolic stress due to mitochondrial function abnormalities (Chance et al., 1981; Pettegrew et al., 1997; Valkovič et al., 2019). One question that emerges from our findings is why the metabolic state would be higher in mild AD than in aMCI, where neurobiological mechanisms compromised status starts? One possibility could be that the higher metabolic state finding in mild AD may represent metabolic stress due to mitochondrial function alterations in the early AD stages. Specifically, we propose that the turnover rate of energy production and utilization represented by energy reserve and energy consumption indices, which are altered as early as in aMCI, may potentially send a cascade of disruptions to neuronal function leading to higher metabolic stress and cognitive decline as the disease is progressing from aMCI and AD.

A pattern of elevated metabolic state in early AD compared to healthy controls is equivocal given other evidence suggesting the opposite relationship between Pi to PCr ratio (Rijpma et al., 2018). Using ^31^P MRS at 3T, Rijpma et al. (2018) found a lower metabolic state in individuals with mild AD versus healthy controls in four brain regions (i) right hippocampus of the temporal lobe (ii) left hippocampus of the temporal lobe (iii) anterior cingulate cortex, (iv) retrosplenial cortex. The discrepancy of the higher metabolic state ratio in this study versus lower in the Rijpma study may be due, in part, to at least two different factors. One possible explanation is that the current study investigated metabolism in the temporal lobes entirety. In contrast, the research by Rijpma et al. (2018) only reported the hippocampus’s findings within the temporal lobes. Obviously, more focal measurements may be more precise, as it is unclear if the entire temporal lobes metabolic state may be compensating for a lower function in a smaller region (Gould et al., 2006). Another possible explanation for the inconsistent results is that PCr increases with age; therefore, adjusting for age in the data analysis is a crucial step that was not accounted for in Rijpma’s study. In the current study to control for age-effects on the phosphate metabolites, all the metabolites were adjusted for age, gender, and education. Moreover, it is to note that Rijpma’s work could motivate efforts toward developing methodologies that allow for BEM markers to be distinguished using lower field MRI strength such as 3T due to these scanners broader availability. To make this possible, further development of radiofrequency (RF) pulses are required so that the higher resolution of metabolites from UHF is measurable using lower magnet strength, e.g., 3-T, for research followed by its clinical application. In sum, we propose that the altered metabolic state indicator may be disrupted, either at a higher or lower level, in mild AD compared to CN and aMCI. More extensive studies are needed to determine if the metabolic state indicator may differentiate aMCI from CN. The small sample size may have precluded such distinctions. The preliminary data support the possibility that alterations in the p-BEM mechanism may contribute to neuronal pathophysiology dysfunction and cognitive decline in AD’s early stages.

Finally, intracellular Mg^2+^ was significantly lower in mild AD compared to aMCI and CN. The present work adds to prior work of post-mortem findings of low Mg^2+^ in the brain’s vulnerable region using an *in vivo* technique. The use of UHF has enabled to have a high resolution of α- and β-ATP signal, which appeared to be an easy-to-fit singlet they are more complicated as doublet and triplet, respectively, at the lower field at 1.5-T and 3T (Das et al., 2020). Therefore, measuring Mg^2+^ levels precisely could add a new dimension to have a comprehensive approach to understand the role of regulatory co-factors on the p-BEM mechanism, a mitochondrial function.

Overall, this study expanded the knowledge of mitochondrial dysfunction theory from a known BEM marker, i.e., metabolic state indicator, to include two more brain markers, specifically energy reserve and energy consumption indices. Moreover, these two novel brain markers suggest differentiating aMCI from CN based on p-BEM dysfunction theory accounted by the alterations in mitochondrial function observed in the *in vitro* post-mortem AD brain. Overall, we summarize that the dysfunction due to mitochondrial function manifested by changes in energy use and consumption may be disrupted before noticeable metabolic stress is measurable in the brain. The work using UHF is in a nascent stage of development and discovery. Nonetheless, the present results support the viability of investigating p-BEM differences using UHF magnetic strength at 7T as a promising new methodology that may help transfer the knowledge on BEM from *in vitro* to the *in vivo* human brain.

### p-BEM-Cognitive Correspondence

Another critical question addressed in this research was the association across the sensitive p-BEM markers of the temporal lobe bilaterally (energy reserve index, energy consumption index, metabolic state indicator, and intracellular Mg^2+^) and cognitive domains of memory, EF, attention, visuospatial skills, and language in the three groups. In CN, a distinct pattern of higher intracellular Mg^2+^ in the temporal lobe was associated with higher performance in the cognitive domains of memory, EF, and attention. On contrary in aMCI and AD, an inverse relationship was observed across the domains of cognition with Mg^2+^. A possible explanation of opposite results in CN versus aMCI and mild AD is that Mg^2+^ is one of the regulatory co-factors that regulates BEM mechanisms in the cell to support cognition. In typical biological conditions, as one could speculate in the CN group, Mg^2+^ acts as a co-factor to regulate enzymes such as creatine kinases of BEM mechanism to support neuronal function and cognition, i.e., a direct association between Mg^2+^ and cognitive performance. However, in the compromised brain of aMCI and AD, wherein prior studies have supported low levels of creatine kinase (David et al., 1998; Aksenov et al., 2000) could not compensate for the BEM mechanism despite adequate Mg^2+^ to support cognition. The present findings do not adequately reveal what is happening due to limitations in measuring creatine kinase activity. Future work is needed to expand on the present evidence to explore a possible role of intracellular Mg^2+^ on cognitive performance across the three cohorts.

### Prediction Model of MRS and Neurocognitive Measures

The final goal was to develop a prediction model for future applications on the extensive data set. From a theoretical perspective, such approaches will test p-BEM distinction validity by seeking to classify the individuals into their respective groups based on these early molecular changes in the brain vulnerable to the disease. Over the past decade, imaging tools like PET-tau and ^18^FDG-PET have offered a clinically relevant methodology to investigate early pathological biomarkers internal to cells and predict who will convert to AD in addition to cognition (Koychev et al., 2017; Ding et al., 2019). Therefore, the prediction model approach in this research may help expand the utility of p-BEM characterization across many populations along with neurocognitive measures to improve early detection and prediction as to who will convert to AD in a longitudinal study.

## Limitations

The results are interpreted cautiously due to several limitations. First, the sample size in each cohort was small, making robust conclusions uncertain. Nonetheless, the data showed a promising pattern of altering phosphate BEM markers, a methodology to explore mitochondrial function in transitory stages even before AD’s full symptomology. Second, a prior study using either fMRI or PET has focused more on identifying brain areas where disease starts. In this study, investigating the brain’s focal vulnerable region was limited due to the larger voxel size. Moreover, the present study reports only relative concentrations of the metabolites in the resting state. Future work is required to be focused on measuring the absolute concentrations of the phosphate metabolites in real-time to understand the rate of turnover of BEM mechanics. Third, as the technological advancements in analyzing partial-volume effects of ^31^P MRS data were limited, in this study, certain regions of the brain on the border with skull or overlapping regions in a given voxel were excluded, thus following a conservative approach for analysis. To overcome this particular issue, in future studies we are rapidly developing new analytical approaches from the ^31^P MRS raw data. Fourthly, brain atrophy was not accounted for in the analysis due to the small sample size. Lastly, while we developed a deep learning model with a small study population, the model may not be a robust model for future application to a more extensive data set of BEM markers and neurocognitive measures.

## Conclusion

To summarize, the present study provides preliminary evidence that phosphate BEM changes may be an early biomarker of AD pathophysiological changes even before the disease symptoms are noticeable. For over a century, AD pathology was associated with the accumulation of β-amyloid and tau, followed by the recent development of glucose metabolism abnormalities contributing to neurodegeneration. However, an unanswered question is if upstream phosphate BEM markers abnormalities cause these known pathological abnormalities cascade. Future directions would further develop this effort to deploy ^31^P MRS in conjunction with PET-amyloid,-tau, and ^18^FDG to investigate how abnormal BEM markers modulate the burden of amyloid, tau, and abnormal glucose metabolism in AD neuropathophysiology. Moreover, a longitudinal study is required for tracking p-BEM markers biological mechanisms changes internal to the cell associated with cognitive decline as individuals move from normal aging to the transitory stage of aMCI and AD.

## Data Availability Statement

The raw data supporting the conclusions of this article will be made available by the authors, without undue reservation.

## Ethics Statement

The studies involving human participants were reviewed and approved by The University of Texas Southwestern Medical Center. The patients/participants provided their written informed consent to participate in this study.

## Author Contributions

ND was the principal investigator who played an integral role in developing and implementing the research protocol followed by data collection, analysis and writing the manuscript. JR has been instrumental in the analysis of MRS data along with ND. JS biostatistician was involved in the statistical analytical approach. SC mentored the writing of the manuscript along with research protocol implementation. All authors contributed to the article and approved the submitted version.

## Conflict of Interest

The authors declare that the research was conducted in the absence of any commercial or financial relationships that could be construed as a potential conflict of interest.

## Abbreviations

xAD: Alzheimer’s disease
aMCI: amnestic mild cognitive impairment
p-BEM: phosphate brain energy metabolism
^31^P MRS: ^31^Phosphorus MRS
ATP: adenosine triphosphate
PCr: phosphocreatine
Pi: inorganic phosphate
PE: phosphoethanolamine
PC: phosphocholine
GPE: glycerophosphoethanolamine
GPC: glycerophosphocholine
Mg^2+^: magnesium
A β: beta-amyloid
UHF: ultra-high-field
ADNI: Alzheimer disease neuroimaging initiative.
