# Supplementary material for: Phosphate Brain Energy Metabolism and Cognition in Alzheimer’s Disease: A Spectroscopy Study Using Whole-Brain Volume-Coil 31Phosphorus Magnetic Resonance Spectroscopy at 7Tesla
Source: Front Neurosci. 2021 Apr 6;15:641739. doi: 10.3389/fnins.2021.641739 (PMC8055842; doi:10.3389/fnins.2021.641739)
Supplement: Supplementary file 1 [file Table_1.DOCX]

**Table 1 ANOVA analysis of neurocognitive measures across the three cohorts: cognitively normal (CN) adults, amnestic mild**

**cognitive impairment (aMCI), and mild Alzheimer’s disease (AD). Tukey multiple comparisons of means (p≤0.05)**

| **Neurocognitive measures** | **Results** | | **Post-hoc Tukey multiple comparisons** | | |
| --- | --- | --- | --- | --- | --- |
|  | F-statistics  (df=2,38) | p-value | MCI to CN mean difference (p-value) | AD to CN mean difference (p-value) | AD to MCI mean difference (p-value) |
| **Executive function** | | | | | |
| 1. **Complex abstraction** 2. TOSL 3. WAIS-Similarities | 1.176  38.87 | 0.319  <0.001*** | -0.29(0.71)  -0.56(0.04) | -0.60(0.29)  -2.02(<0.001***) | -0.32(0.70)  -1.47(<0.001***) |
| 1. **Innovation**   TOSL | 5.04 | 0.01* | -0.31(0.63) | -1.13(0.009**) | -0.82(0.07) |
| 1. **Inhibition and switching**   Trails B | 50.28 | <0.001*** | 0.05(0.96) | 1.93(<0.001***) | 1.87(<0.001***) |
| 1. **Conceptual reasoning**     Delis-Kaplan executive function system (DKEFS) card sort   1. Correct sort 2. Correct description 3. Recognition 4. Composite | 29.9  32.56  24.89  39.12 | <0.001***  <0.001***  <0.001***  <0.001*** | -0.30(0.42)  -0.34(0.30)  -0.43(0.20)  -0.53(0.0477) | -1.87(<0.001)***  -1.91(<0.001)***  -1.84(<0.001)***  -2.02(<0.001)*** | -1.57(<0.001)***  -1.55(<0.001) ***  -1.41(<0.001) ***  -1.50(<0.001) *** |
| 1. **Working Memory**   Digit Span backward test | 3.906 | 0.029 | -0.59(0.21) | -1.02(0.02) | -0.43(0.48) |
| 1. **Fluency**   **Verbal fluency-FAS**  **Category fluency-Animals** | 17.16  10.38 | <0.001***  <0.001*** | -0.13(0.88)  -0.26(0.66) | -1.60(<0.001***)  -1.43(<0.001***) | -1.47(<0.001***)  -1.17(0.003**) |
| **Episodic Memory** | | | | | |
| **1. California verbal learning task-**   1. Immediate recall 2. Short delay recall 3. Long delay recall 4. Intrusions | 26.6  35.82  27.76  8.60 | <0.001***  <0.001***  <0.001***  <0.001*** | -0.83(0.004**)  -0.80(0.002**)  -0.86(0.003**)  0.37(0.46) | -1.92(<0.001***)  -2.03(<0.001***)  -1.93(<0.001***)  1.38(<0.001***) | -1.09(<0.001***)  -1.22(<0.001***)  -1.08(<0.001***) 1.00(0.013) |
| **Attention** | | | | | |
| 1.Selective Auditory Learning Test   1. Trial 1 2. Trial 2 3. Trial 3 | 51.2  9.74  17.05 | <0.001***  <0.001***  <0.001*** | -0.78(<0.001***)  -0.19(0.81)  -0.32(0.48) | -2.14(<0.001***)  -1.38(<0.001***)  -1.66(<0.001***) | -1.36(<0.001***)  -1.89(<0.001***)  -1.34(<0.001***) |
| 2. Digit Span Forward Test | 4.077 | 0.025 | -0.34(0.58) | -1.05(0.02) | -0.71(0.15) |
| **Language** | | | | | |
| 1. Boston Naming Test (BNT) | 22.2 | <0.001*** | -0.00(1.00) | -1.64(<0.001***) | -1.63(<0.001***) |
| **Visuo-spatial skills**  Trails A | 16.79 | <0.001*** | -0.22(0.69) | 1.41(<0.001***) | 1.63(<0.001***) |

**Table 2 General linear model: Estimates of cognitive performance outcome modeled by p-BEM markers- energy reserve index, energy consumption index, metabolic state indicator and regulatory co-factor magnesium (Mg^2+^) and group.**

| **Region of interest BEM interaction with clinical group to model on cognitive outcome** | **Interaction contrasts CN-MCI** | | | | **Interaction contrasts AD-MCI** | | | | **Interaction contrasts AD-CN** | | | | | |
| --- | --- | --- | --- | --- | --- | --- | --- | --- | --- | --- | --- | --- | --- | --- |
|  |  | | | |  | | | |  | | | | | |
|  | **t-value** | **Contrasts of regression coefficients(b4)** | **Standard error** | ***p-value*** | **t-value** | **Contrasts of regression coefficients(b5)** | **Standard error** | ***p-value*** | | **t-value** | **Contrasts of regression coefficients**  **(b6).** | | **Standard error** | ***p-value*** |
| 1. ***Temporal lobe- Energy reserve index (PCr/t_ATP)*** | | | | | | | | | | | | | | |
| Executive function  Verbal fluency  1.A  Inhibition and switching-Trails B | 1.70  -1.18 | 0.54  -0.24 | 0.32  0.21 | 0.10  0.25 | -2.90  3.84 | -1.74  1.50 | 0.60  0.39 | 0.006  0.0005 | -1.98  3.20 | | | -1.20  1.26 | 0.60  0.39 | 0.06  0.003 |
| Visuospatial domain  Trails A | -1.08 | -0.31 | 0.29 | 0.29 | 2.80 | 1.52 | 0.54 | 0.008 | 2.12 | | | 1.21 | 0.55 | 0.03 |
| ***b. Temporal lobe- Energy consumption index (intracellular_Pi/t_ATP)*** | | | | | | | | | | | | | | |
| Executive function  Verbal fluency (A) | 1.60 | 0.49 | 0.30 | 0.12 | -2.36 | -0.90 | 0.38 | 0.023 | -1.01 | | | -0.41 | 0.407 | 0.32 |
| Memory  Episodic memory  California Verbal Learning Task  (CVLT)  1. List A-immediate recall  2.Repetitions | -1.10  -2.48 | -0.28  -0.83 | 0.26  0.33 | 0.28  0.018 | 1.20  -0.45 | 0.36  -0.19 | 0.32  0.42 | 0.27  0.66 | 1.39  -2.26 | | | 0.42  -1.01 | 0.30  0.45 | 0.17  0.03 |
| 1. ***Temporal lobe- Metabolic state indicator (intracellular_Pi/PCr)*** | | | | | | | | | | | | | | |
| Executive function  Innovation-TOSL | 2.68 | 1.29 | 0.48 | 0.01 | 0.06 | 0.02 | 0.34 | 0.95 | 2.94 | | | 1.31 | 0.44 | 0.006 |
| Memory  Episodic memory  California Verbal Learning Task (CVLT)  1.List A- Immediate memory recall  2.Recognition | -2.38  -2.65 | -0.98  -1.28 | 0.41  0.48 | 0.02  0.011 | 0.91  3.08 | 0.27  1.06 | 0.30  0.34 | 0.38  0.004 | -1.86  -0.49 | | | -0.71  -0.22 | 0.38  0.45 | 0.07  0.63 |
| ***4. Temporal lobe -Regulatory factors: intracellular magnesium (Mg^2+^)*** | | | | | | | | | | | | | | |
| Executive function  Complex abstraction-TOSL | -2.38 | -1.41 | 0.60 | 0.023 | 0.22 | 0.09 | 0.39 | 0.82 | -2.39 | | | -1.32 | 0.55 | 0.02 |
| Episodic memory  California Verbal Learning Task  (CVLT)  List A-immediate recall | -2.62 | -1.02 | 0.39 | 0.013 | 0.52 | 0.13 | 0.26 | 0.61 | -2.45 | | | -0.89 | 0.36 | 0.02 |
| Attention  Strategic Auditory attention test  1.Trail 1  2.Trail 2 | -3.99  -2.67 | -1.14  -1.31 | 0.29  0.49 | 0.0003  0.01 | 1.28  1.66 | 0.24  0.54 | 0.19  0.32 | 0.21  0.11 | -3.39  -1.69 | | | -0.90  -0.78 | 0.27  0.46 | 0.002  0.098 |

**Figure 1 Schematic representation of principal component reduction of both neurocognitive measures and BEM markers combined in the whole data set.**
